# Influence of gastric residual assessment in preterm neonates on time to achieve enteral feeding (the GRASS trial)—Multi-centre, assessor-blinded randomised clinical trial

**DOI:** 10.1007/s00431-024-05483-w

**Published:** 2024-03-01

**Authors:** A Branagan, C Murphy, A O’Sullivan, I Bodnarova, S Feyereislova, I Berka, J Miletin, Z Stranak

**Affiliations:** 1 The Coombe Women and Infants University Hospital, Dublin 8, Ireland; 2 Institute for the Care of Mother and Child, Prague, Czech Republic; 3 3rd School of Medicine, Charles University, Prague, Czech Republic; 4 UCD School of Medicine, University College Dublin, Dublin, Ireland; 5 2nd Faculty of Medicine, Motol University Hospital, Prague, Czech Republic

**Keywords:** Prematurity, Very low birth weight, Gastric residual, Necrotising enterocolitis

## Abstract

**Purpose:**

Gastric residual measurement is routinely performed in premature infants prior to feeding despite a lack of evidence of benefit. We aimed to evaluate if the exclusion of routine gastric residual measurement and evaluation has an impact on the time taken to achieve full enteral feeding in preterm neonates.

**Methods:**

International multi-centre randomised controlled trial. Clinically stable, appropriate for gestational age infants between 26^+0^ and 30^+6^ weeks of gestation and less than 1.5 kg birth weight were eligible. Infants were randomised to the intervention arm (no monitoring of gastric aspirates) or control arm (routine care). Primary outcome was the achievement of enteral feeds of 100 ml/kg/day by day 5 of life.

**Results:**

Ninety-five infants were recruited with 88 included in an intention-to-treat analysis, 45 in the intervention arm and 43 in the control arm. There was no imbalance in baseline characteristics. Thirty-three (73.3%) infants in the intervention group and 32 infants (74.4%) in the control group reached full feeds by day 5 of life (*p* = 0.91) with no difference in median time to full feeds. There were no statistically significant differences in survival or the major morbidities of prematurity.

* Conclusion*: There was no difference in time to attainment of enteral feeds of 100 ml/kg/day in premature infants when gastric residuals were not monitored. In the absence of a clinical benefit to routine monitoring, it may be appropriate to discontinue this practice and only monitor residuals when clinical concern of feeding intolerance or gastrointestinal pathology arises in this group of patients.

*Trial registration*: NCT03111329—https://clinicaltrials.gov/. Registered 06/04/2017.

**Table:** 

**What is Known:** *• Previous randomized trials have shown little benefit to the performance of routine assessment of gastric residuals in preterm infants. Despite this, they continue to be performed due to concerns from observational data regarding development of NEC. Meta-analysis to date has failed to answer the question regarding NEC.*	
**What is New:** *• In very low birth weight infants who are fed using modern feeding practice of faster feed advancement, to minimize use of central access and parenteral nutrition, exclusion of routine checks of gastric residuals did not increase the proportion of infants reaching full enteral feeds by day 5. No harm was seen when residual checks were not performed.* *• In the absence of a clinical benefit to the routine performance of gastric residuals in very low birth weight infants, it may be appropriate to discontinue their use and instead check residuals when clinical concern of pathology arises.*

## Introduction

Gastric residual measurement, the aspiration and evaluation of stomach contents, is routinely performed prior to feeding preterm infants in the neonatal intensive care unit (NICU) [1, 2]. Despite a lack of evidence confirming benefit, aspirates are used extensively to confirm naso- or orogastric (NG/OG) tube placement, monitor the amount and appearance of previous feed remaining in the stomach and prevent aspiration of stomach content. Residuals are used as an indicator of feeding intolerance. Feed intolerance occurs in preterm infants for a variety of non-pathological reasons including reduced motility and the normal structural and functional immaturity of the gastrointestinal (GI) tract [3–5]. Historically, large or bile-stained residuals have been seen as an indicatoro of developing necrotising enterocolitis (NEC) [6, 7]. However, there is also potential harm associated with routine checks—loss of necessary gastric enzymes and micronutrients and mucosal irritation and damage [8]. Because of concern about the potential relationship between residuals and GI pathology, their presence may lead to the holding of feeds or delays in advancing volumes, which can in turn delay the removal of central access.

Adequate nutrition in the early neonatal period is a vital factor in the management of very low birth weight (VLBW) infants (birth weight < 1500 g). While nutrition can be provided via parenteral nutrition (PN), extending the requirement for PN, and therefore, intravenous (IV) access can be associated with increased rates of late-onset sepsis (LOS) [9, 10]. Appropriate early extrauterine growth has been associated with fewer medical complications in the neonatal and infant period [11, 12], improved neurodevelopmental outcomes [13, 14] and decreased metabolic complications in later life [15, 16]. Therefore, establishing enteral feeding promptly yet safely is paramount [17].

To date, multiple randomised and observational studies have evaluated the impact of gastric residuals. Although varying results are described, little evidence of benefit has been found. Our aim was to investigate if the exclusion of routine checks of gastric residuals would have an impact on the time taken to achieve enteral feeding of 100 ml/kg/day, in VLBW neonates.

## Methods

### Study design

An international multi-centre randomised controlled trial was carried out in two level III NICUs (The Coombe Women and Infants University Hospital (CWIUH), Dublin, Ireland, and the Institute for the Care of Mother and Child (ICMC), Prague, Czech Republic) between 6th October 2017 and 12th April 2021. Trial protocol was registered before commencement (NCT03111329—https://clinicaltrials.gov/). Ethical approval was granted at each site (CWIUH REC 3-2019 approved on 27/03/2019, ICMC REC EK-2017-01-05 approved on 19/12/2016). Written informed consent was obtained prior to enrolment, antenatally where possible, or in the first 6 h of life and before the infant received their first enteral feed.

### Participants

VLBW infants born at a gestational age (GA) between 26^+0^ and 30^+6^ were eligible for inclusion. Exclusion criteria were intrauterine growth restriction (birth weight below 5th centile for GA and gender on World Health Organisation [18] or intergrowth [19] charts), life-threatening events requiring full resuscitation in the delivery room with a persistently raised lactate of more than 5 mmol/l, circulatory instability requiring treatment with inotropes, highly suspected early-onset sepsis with alteration of general clinical state (worsened peripheral perfusion and circulatory decompensation during the first 6 h of life) and known malformations of GI tract or any other life-limiting serious congenital malformations.

### Randomisation and masking

Computer-generated randomisation was performed with random permuted blocks of 8, to assign infants in a 1:1 allocation ratio to the intervention arm (no monitoring of gastric residuals) or control arm (usual care). Randomisation was stratified by gestational age and centre. Twins were assigned independently. Randomisation lists were prepared by a research coordinator with no clinical involvement in the trial. Group assignment was concealed within sealed, opaque, sequentially numbered envelopes, stored centrally in each unit. After infants were stabilised in NICU with central venous access, via an umbilical venous catheter or peripherally inserted central catheter, the next envelope in the sequence was opened to determine group assignment. Due to the nature of the intervention, caregivers could not be blinded. Outcome assessors were blinded.

### Study procedure

Feeding protocols were the same in both centres. Infants had central access inserted on admission and were commenced on PN. Enteral feeds were commenced via NG or OG tube when maternal expressed breast milk (EBM) was available, up to 30 ml/kg/day for the first 24 h and increasing by 30 ml/kg/day until a target volume of 150 ml/kg/day was achieved. If sufficient maternal EBM was not available, donor milk was commenced with the same protocol for progression. All infants were treated with a probiotic (ProPrems^®^, NeoBiomics, Sweden) which was commenced with first enteral feed and discontinued at 34 weeks corrected gestational age. Fortification was commenced at 100 ml/kg/day with cow's milk-based fortifier (SMA^®^ Gold Prem Breast Milk Fortifier, SMA Nutrition, Nestle) which was discontinued at 6 weeks corrected age. Central access was removed when an infant reached 120 ml/kg/day of enteral feeding [20].

Infants were randomised to either control or intervention group. Infants in the control group were managed using routine care. Infants were fed every 2–3 h. NG tube placement was confirmed with an acid reaction on litmus paper. Gastric content was aspirated prior to every feed. Acceptable volume was defined as one-third of the 6-hourly volume of feed. Infants with an unacceptable residual volume were deemed to have potential feed intolerance. Infants in the intervention group were managed without gastric residual aspiration. NG tube placement was confirmed every 6 h by aspirating a minimal amount (0.1 ml) of gastric content. ‘Venting’ of the NG tube, solely opening the tube to air to relieve possible backflow of gastric content, was allowed once every 6 h for 30 min. Tolerance of feed was assessed clinically.

A cross-over rescue strategy was pre-defined for the failure of the intervention arm as a safety criterion. If an infant in the intervention arm failed the intervention arm—defined as the withholding of enteral feeds for 6 h or more due to clinical concern of feed intolerance or GI pathology—they were treated as per the control arm for the remainder of the trial—i.e. regular gastric residual checks were performed.

For infants in both groups, feed intolerance was defined as one of (1) profound vomiting (defined as the forceful emission of gastric contents from the mouth), (2) bilious vomiting, (3) severe abdominal distention with visible bowel loops, (4) tender abdomen on palpation, (5) backflow of bilious gastric contents from a freely open NG tube and (6) gastric residual greater than 1/3 of total feed volume in the control arm. Possible interventions in the case of feed intolerance in both groups were at the discretion of the treating physician: (1) withhold enteral feeding entirely, (2) reduce the amount of enteral feed by 50% and (3) maintain feed volume at the current level.

### Study end points

Primary outcome is the achievement of enteral feeds of 100 ml/kg/day, by day 5 of life. Secondary outcomes are (1) time taken to reach enteral feeds of 100 ml/kg/day, (2) withholding of enteral feeds during the hospital stay, (3) total duration of PN, (4) total duration of an indwelling central venous and arterial catheter, (5) incidence of LOS (defined by KISS criteria) [21], (6) incidence of medical and surgical NEC (defined by Bell’s staging and requirement of surgical treatment) [22], (7) incidence of spontaneous intestinal perforation (SIP), (8) incidence of chronic lung disease (defined as oxygen requirement or need for respiratory support at 28 days of life) and bronchopulmonary dysplasia (defined as oxygen requirement or need for respiratory support at 36 weeks corrected gestational age), (9) incidence of intra-ventricular haemorrhage, (10) incidence of retinopathy of prematurity and (11) hypoglycaemia after the achievement of full enteral feeds.

### Sample size calculation

Sample size was based on a retrospective review of feeding practice in one centre (ICMC). It was estimated that 65% would achieve 100 ml/kg/day of enteral feeds by day 5 of life. To demonstrate a clinically important increase in the primary outcome to 90% (25% increase), we calculated that 43 infants were required in each group with 80% power and an α of 0.05 (chi-squared test).

### Statistical analysis

Data was collected in Excel (Microsoft, USA), and intention-to-treat analysis was performed using StatsDirect v.3.3.5 (StatsDirect Ltd, Cheshire, UK). We compared normally distributed continuous variables with *t*-tests and compared dichotomous and categorical variables with non-parametric tests (*χ*^2^ test, Mann-Whitney *U* test, Fisher’s exact test). Data is presented as median (interquartile range (IQR)), mean (standard deviation (SD)) and 95% confidence intervals (CI) as appropriate.

## Results

### Enrolment

**Fig. 1:**
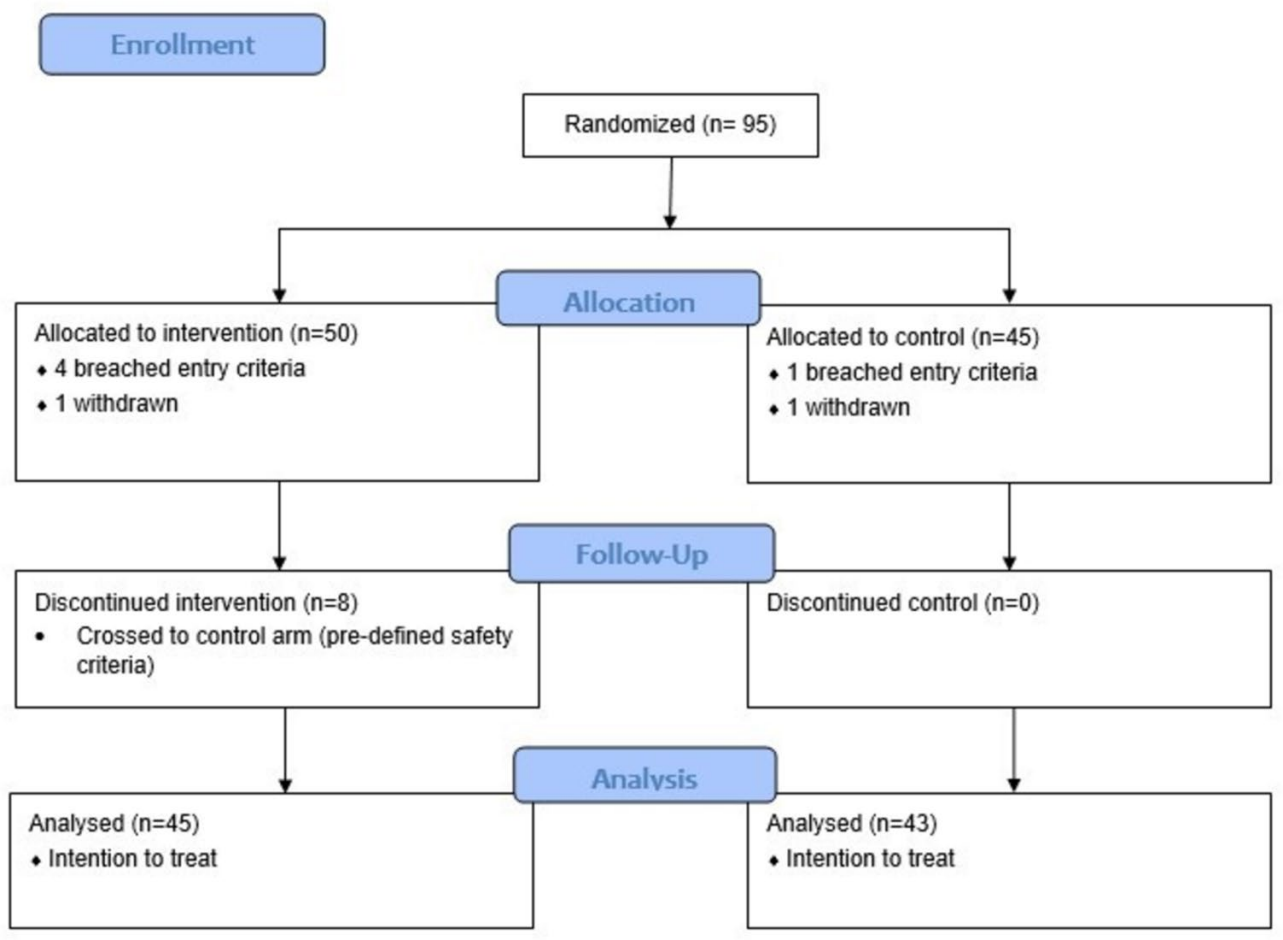
Consort flow diagram

Patient flow is detailed in Fig. 1. Ninety-five infants were recruited between October 2017 and April 2021. Seven infants were excluded—parental consent was withdrawn for two infants, five breached criteria for study entry—two had birth weight ≥ 1.5 kg and three met criteria for IUGR.

Eighty-eight infants were included final analysis, 45 in the intervention arm and 43 in the control arm.

### Patient characteristics

Patient characteristics between groups were similar at study entry (Table 1). In the intervention arm, mean gestational age (SD) was 29 (2) weeks of gestation with a mean birth weight (SD) of 1143 g (241). In the control group, mean gestation (SD) was 29 (1) weeks with a mean birth weight (SD) of 1159 g (270). Time of the first enteral feed varied widely but was similar between groups.

**Table 1:** Baseline characteristics

	**Intervention (*n* = 45)**	**Control (*n* = 43)**
Gestational age, mean (SD) (weeks)	29 (2)	29 (1)
Birth weight, mean (SD) (grams)	1143 (241)	1159 (270)
Birth weight < 1000 g, *n* (%)	15 (33)	13 (30)
Birth OFC, median (25th, 75th quartile) (cm)	27 (25, 28)	27 (26, 28)
Gender, *n* (%), boy	22 (49)	19 (44)
Singleton, *n* (%)	32 (71)	27 (63)
Antenatal steroids		
* n* (%) 2 doses	36 (80)	28 (65)
* n* (%), 1 dose	9 (20)	15 (35)
* n* (%), no steroid	0 (0)	0 (0)
Delivery mode		
SVD, *n* (%)	10 (22)	10 (23)
Caesarean delivery, *n* (%)	35 (78)	33 (77)
Arterial cord pH, median (25th, 75th quartile)	7.32 (7.25, 7.36)	7.33 (7,22, 7.33)
Apgar score at 5 min, median (25th, 75th quartile)	9 (8, 9)	9 (8, 9)
Surfactant ≥ 1 dose, *n* (%)	18 (40)	24 (56)
Early-onset sepsis (within 72 h of birth), *n* (%)	3 (7)	0 (0)
Age at first enteral feed, median (25th, 75th quartile, IQR) (hours)	11 (7, 18)	14 (5, 23)
Feeding (day 1–5)		
Maternal breast milk only, *n* (%)	8 (18)	7 (16)
Donor breast milk only, *n* (%)	1 (2)	3 (7)
Mixed maternal/donor milk, *n* (%)	36 (80)	33 (77)

*SD* standard deviation, *OFC* occipito-frontal circumference, *SVD* spontaneous vaginal delivery

### Primary and feeding outcomes

All infants survived to the primary endpoint. Regarding the primary outcome, 33 (73%) infants in the intervention group and 32 (74%) infants in the control group reached enteral feeds of 100 ml/kg/day by day 5 (*p* = 0.91) (Table 2). Median time (IQR) to feeds of 100 ml/kg/day was 111 (17) h in the intervention arm compared to 111 (24) h in the control arm (*p* = 0.99).

**Table 2:** Primary and feeding outcomes

	**Intervention (*n* = 45)**	**Control (*n* = 43)**	***P* value**
Feeds of 100 ml/kg at 5 days, n (%)	33 (73)	32 (74)	0.91
Survival to primary endpoint, *n* (%)	45 (100)	43 (100)	N/A
Time to feeds of 100 ml/kg, median (25th, 75th quartile) (hours)	111 (99, 116)	111 (96, 120)	0.99
Failed Intervention arm, *n* (%)	8 (18)	N/A	N/A
Ever NPO, *n* (%)	16 (36)	9 (21)	0.14
Duration of NPO, median (25th, 75th quartile) (hours)	10 (8, 48)	10(5, 19)	0.47

*NPO* no enteral feeding, *N/A*not applicable

A subgroup analysis of extremely low birth weight infants (< 1000 g) also showed no difference in the primary outcome.

Sixteen infants (35.6%) in the intervention group and 9 infants in the control group (20.9%) had feeds held at least once during NICU admission with no difference in duration of withholding.

Eight infants in the intervention group (17.8%) failed the intervention arm (enteral feeds withheld for > 6 h).

### Survival and co-morbidities

Survival and co-morbidities were similar between groups (Table 3).

**Table 3:** Survival and co-morbidities

	**Intervention (*n* = 45)**	**Control (*n* = 43)**	***P* value**
Survival to discharge or transfer, *n* (%)	42 (93)	42 (98)	0.39
Discharge gestation, median (25th quartile, 75th quartile, IQR) (weeks)	36 (35.3, 37.4)	36.4 (35.9, 37.9)	0.50
Length of stay, mean (SD) (days)	47.5 (23)	49 (20)	0.50
Discharge weight, mean (SD) (kg)	2.22 (0.7)	2.31 (0.5)	0.46
Discharge OFC, mean (SD) (cm)	32 (2)	32 (2)	0.42
Postnatal steroids, *n* (%)	9 (20)	3 (7)	0.09
Time PICC line in situ, median (25th, 75th quartile) (days)	5.5 (5, 7) *n* = 38	5 (5, 6) *n* = 37	0.71
Time UVC in situ, median (25th, 75th quartile) (days)	3 (2, 6) *n* = 15	3 (2, 6) *n* = 11	0.64
Time UAC in situ, median (25th, 75th quartile) (days)	3 (2,6) *n* = 14	3 (2, 6) *n* = 9	0.54
TPN duration, median (25th, 75th quartile) (days)	6 (5, 7)	6 (5, 6)	0.34
Patent ductus arteriosus			
Medical management, *n* (%)	3 (7)	3 (7)	0.96
Surgical management, *n* (%)	0 (0)	0 (0)	
Necrotizing enterocolitis			
≥ 2A Bells staging, *n* (%)	3 (7)	0 (0)	0.13
Requiring drain or surgery, *n* (%)	2 (4)	0 (0)	0.26
Spontaneous intestinal perforation, *n* (%)	0 (0)	1 (2)	0.49
Late-onset sepsis (> 72 h), *n* (%)	9 (20)	5 (12)	0.30
Interventricular haemorrhage			
Any IVH, *n* (%)	9 (20)	4 (9)	0.17
Grade 3 or 4 IVH, *n* (%)	0 (0)	1 (2)	0.49
Chronic lung disease (of survivors)			
O_2_/respiratory Support at 28 days, *n* (%)	14 (33)	14 (33)	> 0.99
O_2_/respiratory Support at 36 weeks CGA, *n* (%)	8 (19)	5 (12)	0.39
Ventilation			
Required MV, *n* (%)	16 (36)	13 (30)	0.65
Duration of MV, median (25th, 75th quartile) (hours)	45 (23, 192) (*n* = 16)	20 (6, 60) (*n* = 13)	0.13
Hypoglycaemia (< 2.5 mmol/l) after enteral feeds,* n* (%)	3 (7)	2 (5)	0.72
ROP requiring treatment, *n* (%)	1 (2)	1 (2)	0.98

*SD* standard deviation, *OFC* occipito-frontal circumference, *PICC* peripherally inserted central catheter, *UVC* umbilical venous catheter, *UAC* umbilical arterial catheter, *TPN* total parenteral nutrition, *IVH* intra-ventricular haemorrhage, *MV* mechanical ventilation, *ROP* retinopathy of prematurity

Three infants in the intervention arm and one in the control arm died prior to discharge (*p* = 0.39). There was no difference between the groups in discharge gestation, length of stay or discharge weight. There was no difference in the requirement for IV access or PN. Regarding complications of prematurity, there were no statistically significant differences seen.

Three infants (6.7%) in the intervention group developed NEC ≥ 2A on Bells staging with two of these requiring surgery. No infant who developed NEC failed the intervention, and all became unwell after reaching the primary outcome successfully. No infant in the control group developed NEC.

## Discussion

In this trial, we found no statistically significant difference in the establishment of enteral feeds of 100 ml/kg/day of expressed breast milk, by day of life five, in a group of appropriately grown VLBW infants born between 26^+0^ and 30^+6^ weeks of gestation. There were no differences in prespecified secondary outcomes.

Previous literature in this area has concentrated on outcomes regarding feeding achievement and growth rather than safety outcomes such as mortality, NEC, and LOS, leading to meta-analyses to attempt to answer these questions.

The latest Cochrane review on this topic [23] provided a meta-analysis of studies evaluating the measurement of gastric residuals vs. no residuals. Four studies involving 334 infants were included in this comparison. Three studies included infants < 1500 g, while one allowed infants up to 2 kg. They were unable to do a planned subgroup analysis of VLBW infants due to the data available. They found that routine monitoring of gastric residuals probably has little or no effect on the risk of NEC with moderate certainty of evidence (RR 1.08, 95% CI 0.46 to 2.57). They probably increase time to full feeds, TPN days and risk of invasive infection with little or no difference in all-cause mortality predischarge. They conclude that further trials are warranted to increase certainty and assess longer-term outcomes.

This review included the first randomised trial of residual assessment [8] of 61 infants under 32 weeks and less than 1.25 kg, which found that infants reached feeds of 150 ml/kg/day and had IV access removed almost 6 days earlier if residuals were not checked. This result did not reach statistical significance but was considered potentially clinically significant. Feeding was notably slower than in our cohort, with a mean (SD) time to 120 ml/kg/day of enteral feeding of 16.8 (12.4) days and 14.3 (12.5) days in the control and intervention arms respectively.

This was followed by a similar trial [24] of 143 infants < 1.25 kg and < 32 weeks randomised to gastric residual assessment or no residual assessment, evaluating the effect of checking residuals on weekly enteral nutrition over the first 6 weeks of life. The no-residual group advanced feeding quicker with higher mean weight gain. They were discharged home 8 days earlier with a similar odds ratio for NEC, LOS and death.

While our results are similar to previous trials in failing to show harm if residuals are not checked, although none have been powered for these outcomes, we did not show any statistical or clinical benefit to their avoidance. This may be explained by the difference in feeding practices between units and the evolution of feeding practices over time. In our study, enteral feeding was commencement earlier, with more rapid advancement and earlier achievement of full enteral feeding, consistent with changes in premature feeding guidelines internationally. Quicker establishment of enteral feeds may have meant less impact of residual measurement, being more difficult to reverse enteral feeding to IV in a baby who is fully established on feeds without IV access readily available. Our cohort was a more mature cohort with larger infants, as we had a higher GA and weight cut-off.

Our protocol allowed clinicians to make pragmatic clinical decisions on the management of what they deemed a concerning aspirate and to combine this with the full clinical picture. This highlights the minimal impact routine measurement has on decision-making in NICU. We allowed regular venting of the gastric tube. As most infants were managed on nasal continuous positive airway pressure, this may have decreased episodes of abdominal distension compared to other studies—leading to less diagnosis of feed intolerance and interruption of advancement.

The main concern regarding the removal of routine checks of residuals is the removal of an early indicator of NEC, removing opportunities to intervene early and minimise morbidity. This is based on the results of two older case–control studies on NEC risk factors [6, 7]. The first [6] suggested that a volume over 3 ml may be associated with a higher risk of NEC. A study of 34 infants [7] observed higher volume residuals in the group which later developed NEC (7.5 ml vs. 4 ml). However, a 17-day delay was noted from maximum residual to a diagnosis of NEC, minimising clinical utility.

While considered a benign investigation, gastric residuals contain essential gastric enzymes and micronutrients which are lost if the aspirate is discarded. Hydrochloric acid is essential to the intestinal barrier and necessary to limit bacterial overgrowth. Discarding aspirates may increase inflammation and further alter the preterm gut microbiome, increasing the risk of late-onset sepsis [25, 26]. Refeeding may be done but remains controversial.

The strengths of this study include the randomisation process with stratification, the multi-centre nature and powering to a clinically relevant endpoint. Our findings are limited to the population we studied and may not be generalizable to infants < 26 weeks, with IUGR or critically unwell infants. As with previous studies, we were not powered to detect a difference in NEC.

## Conclusion

In the absence of a clinical benefit to routinely monitoring gastric residuals in preterm infants, it may be appropriate to discontinue this practice and instead only monitor residuals when clinical concern of feed intolerance or gastrointestinal pathology arises.

## Data Availability

No datasets were generated or analysed during the current study.

## Abbreviations

AC: Abdominal circumference
CWIUH: The Coombe Women and Infants University Hospital
EBM: Expressed breast milk
GA: Gestational age
GI: Gastrointestinal
ICMC: Institute for the Care of Mother and Child
IQR: Interquartile range
IV: Intravenous
IVH: Intra-ventricular haemorrhage
LOS: Late-onset sepsis
MV: Mechanical ventilation
NEC: Necrotising enterocolitis
NG: Nasogastric
NICU: Neonatal intensive care
NPO: No enteral feeding
OFC: Occipito-frontal circumference
OG: Orogastric
PICC: Peripherally inserted central catheter
PN: Parenteral nutrition
ROP: Retinopathy of prematurity
SD: Standard deviation
SIP: Spontaneous intestinal perforation
SVD: Spontaneous vaginal delivery
UVC: Umbilical venous catheter
VLBW: Very low birth weight
